# Dried-Fruit Storage: An Analysis of Package Headspace Atmosphere Changes

**DOI:** 10.3390/foods8020056

**Published:** 2019-02-04

**Authors:** Gonzalo Miranda, Angel Berna, Antonio Mulet

**Affiliations:** 1 Chemical Engineering Department, University of València, Avenida de la Universitat s/n. 46100 Burjassot, València, Spain; Angel.Berna@uv.es; 2 Department of Food Technology, Polytechnic University of València, Camino de Vera s/n. 46022 València, Spain; amulet@tal.upv.es

**Keywords:** food packaging, food quality, fruit storage, modified atmosphere packaging, permeability

## Abstract

The quality of packaged dried foods depends on storage conditions and is determined largely by the initial gas composition inside and the transference through the container. The aim of this work was to analyze the O_2_ and CO_2_ concentrations within the internal atmosphere of the packaging. In this study, dried apricots and raisins were packaged in glass jars and polypropylene trays thermosealed with different polymers, and stored at 5, 15, 25, and 35 °C. Some trays were flushed with nitrogen just before sealing. In addition, the work relates to other previous papers to investigate the effect of these gases and packages on the stored products, and compares the influence of permeable and impermeable containers on food quality parameters. When packages were flushed with nitrogen before sealing, the O_2_ level in the headspace increased until the outside O_2_ concentration was reached. The CO_2_ concentration increased over time, regardless of the initial atmosphere. Nitrogen had a great influence on the concentration of O_2_, but not on that of CO_2_. Finally, this paper shows that the films and initial gas used in this study had no significant effect on the quality of the stored dried fruit.

## 1. Introduction

Drying is one of the most ancient methods used to preserve foods. Archeological sites show that this technique was used in Egypt and Mesopotamia since 4000 before Christ (BC). Controlling the initial water content and its variation during storage is crucial to maintaining the quality of the product [1]. Due to their significantly active surface, dried fruits and vegetables represent a unique challenge in terms of food protection and are very sensitive to humidity and oxidation during their storage. This is why it is especially important to select the ideal packaging and storage methods in order to prevent these undesirable physicochemical processes, which are detrimental to the products’ quality. The principal aims of the packaging material and internal atmosphere are to keep the food in good condition until it is consumed.

Many studies were conducted with different fruits for the purposes of preserving, storing, and packaging processed products to increase their shelf life [2,3,4]. The effects of packaging materials, storage time, and temperature are critical factors for preserving fruits during their off-season [5]. In order to serve as packaging for dehydrated fruits and vegetables, the material must prove itself to be a good barrier against water vapor and, depending on the particular product, also against O_2_, SO_2_, and other volatiles. The headspace inside the package and the permeability to oxygen and other gases of the material determine the quantity of oxygen permeating into the package during the storage period [6]. 

Consumer research consistently indicates that consumers often perceive glass-packaged products to be of a high quality [7]. Colorless glass used for food packaging is derived from soda, lime, and silica. The main physical advantage of glass is its inertness and impermeability. Moreover, regarding environmental aspects, glass is reusable and recyclable. In recent years, there was a tendency to replace glass with plastic for packaging, as glass, while being excellent at protecting food quality, takes up more space in the store and can break. One of the reasons for the adoption of plastic is the potential environmental benefit of shipping lighter containers, which reduces transport costs [8].

Both polyethylene and polypropylene offer a successful combination of flexibility (both rigid and flexible), strength, lightness, impermeability, stability, etc. Plastic food packaging provides an effective barrier that ensures that food keeps its natural taste while protecting it from contamination. The use of both flexible and rigid polymeric packaging is currently growing rapidly and is driven by new developments in bio-plastics and the desire to reduce the bulk and weight of metal and glass containers [9]. Innovative packaging techniques such as modified active packaging, active and intelligent packaging, the use of antimicrobials, etc. extend the shelf life of fruits to a significant amount to time. The most important active packaging systems involve the use of oxygen scavengers, humidity and ethylene absorbers, carbon dioxide and ethanol emitters, and antimicrobial active packaging systems. Intelligent packaging devices include biosensors, as well as time–temperature, oxygen, carbon dioxide, and microbial growth indicators, etc. [10]. 

Important physicochemical processes that affect the headspace occur during the storage of fruit, which are determined by storage conditions. The internal atmosphere, often due to its oxygen content, is responsible for most food spoilage. Furthermore, the rate of biological reactions generally increases two-or threefold for every 10 °C rise in temperature within the range normally encountered during the storage and distribution of food [11]. The chosen material for the film plays an important role in allowing, or not, the permeation of gases that may accelerate the deterioration of the foodstuff. In addition, at least 2500 volatile compounds are formed as a result of Maillard reactions [12]. Oxygen contact also has an impact on the products of the Maillard process [13]. Thus, maintaining the optimum composition of the headspace by selecting the best packaging material is essential for controlling these various processes.

Great efforts were made in order to maintain the quality of fresh fruits at an optimum level during their entire storage period. Currently, post-harvest treatments and alternative technologies are widely used [14]. Modified atmosphere packaging (MAP), temperature, film composition, and food quality factors were rigorously studied [15,16,17]. The technology of MAP is based on the alteration of the surrounding atmosphere of the product by varying the concentration of gases such as carbon dioxide, nitrogen, and/or oxygen [18]. To date, the literature includes many research works and review articles dealing with the modification of the package atmosphere in order to extend the food shelf life [19,20]. Nitrogen inhibits the growth of aerobic organisms and, consequently, increases the shelf life of food products [2]. 

Although dried fruits are inherently less perishable than fresh ones [21], product spoilage always occurs [22]; consequently, further research into modifying package atmosphere may help improve their shelf life. However, there are not many papers about MAP applied to dried-fruit storage. Ranđelović et al. [4] investigated the influence of the protective properties of the packaging and modified atmosphere on the quality of dried apricots kept at room temperature (17 to 22 °C). They used four different combinations of polyethylene with a layer of another material.

In the present work, in addition to different polymeric combinations, glass was also used as a packaging material in order to compare storage in systems that allow, or not, the exchange of mass with the surroundings. Therefore, this paper is framed in the field of MAP applied to dried fruit and also analyzes the effect of the barrier properties of the package on the internal atmosphere, using, in addition, two types of dried fruit: apricots and raisins. As a hypothesis, the oxygen concentration in the pack was expected to reach an equilibrium with the air outside, since the films used were semipermeable.

The objective of the present work was to evaluate the influence of storage conditions, specifically packaging materials and temperature, on the changes in the headspace composition. This study references and builds on previous research into the changing properties of dried-fruit products themselves, drawing subsequent conclusions and comparisons between how these new findings are interrelated with previous papers on the same fruits [3,23,24]. 

## 2. Materials and Methods

### 2.1. Material Treatment and Drying Processes

This study was carried out with apricots (*Prunus armeniaca* Canino) harvested in June and raisins (*Vitis vinifera* Moscatel Romano) harvested in September, from Valencia and Alicante (Spain), respectively. After being harvested, the apricots were halved and stoned. Both fruits were pre-treated before drying [25]. Halved apricots were immersed in potassium metabisulfite 3% (*w*/*v*) at 40–49 °C for 35 min and then 5 min in acetic acid 1% (*w*/*v*) at 25 °C. Raisins were dipped for 20 s in sodium hydroxide 0.6% (*w*/*v*) at 100 °C, followed by a 5-min immersion in water at 25 °C, then 10 min in potassium metabisulfite 4% (*w*/*v*) at 25 °C and, to finish, 5 min in acetic acid 1% (*w*/*v*) at 25 °C.

Drying was carried out in a pilot-scale convective dryer equipped with a 2-kW ventilator, electric resistances with a total power of 22 kW, and six metal mesh trays with a maximum weight of 60 kg [26]. Samples were placed in a single layer in each tray. The forced hot air circulated at 5 m/s. The apricots were dried at 50 °C and raisins at 40 °C, both for 9 h. To ensure product homogeneity, samples were stored after drying in sealed containers at 10 °C for 72 h. 

At the time of packaging, the dried apricots had 26.6 ± 0.7 g water/100 g dry solid, 0.56 ± 0.09 g SO_2_/kg dry solid, and a texture of 38 ± 8 N/cm^2^. Raisins had 26.8 ± 0.1 g water/100 g dry solid and 0.37 ± 0.02 g SO_2_/kg dry solid.

### 2.2. Packaging and Storage

Subsequently, one set of samples of the dried apricots and raisins was stored in 350-cm^3^ glass containers with metal twist-off caps and another set was stored in 500-cm^3^ (158 × 112 mm open face) polypropylene trays supplied by Orved S.L. (Barcelona, Spain) and thermosealed with transparent films (the only gas exchange surface) supplied by Südpack España S.L. (Barcelona, Spain). These films were as follows:

Oriented polyamide/polyethylene (OPA/PE) 15/100: a 15-mm-thick OPA layer with a 100-mm PE layer;
Polyamide/polypropylene (PA/PP) 20/75: a 20-mm-thick PA layer with a 75-mm PP layer;
Polyamide/polyethylene (PA/PE) 20/70: a 20-mm-thick PA layer with a 70-mm PE layer.

Every package was filled with 74 ± 2 g of sample at room conditions (22 °C and 70% relative humidity (RH)), occupying a volume of 91 ± 6 mL. The trays were sealed mechanically with a device equipped with a vacuum pump and a system to inject different gases (Ilpra, model Food Pack, Barcelona, Spain).

The sets were as follows:
Dried apricots: glass container with air, film OPA/PE 15/100 with air, film OPA/PE 15/100 with nitrogen, and film PA/PP 20/75 with air;
Raisins: glass container with air, film PA/PP 20/75 with air, film PA/PP 20/75 with nitrogen, and film PA/PE 20/70 with air.

Each storage condition was replicated 10 times. Analyses of the films were carried out to confirm composition and thickness and to determine their permeability to O_2_, CO_2_, and water vapor. Moreover, the atmosphere inside the polypropylene packages and the parameters of food stored both in polypropylene and in glass containers were analyzed periodically throughout the 12-months storage. In the case of raisins, these analyses were at 14, 84, 126, 190, 251, and 343 days, and, in the case of apricots, they were at 13, 21, 87, 125, 193, 245, 300, and 352 days.

Table 1 shows the properties of the films used to seal the polypropylene trays. Permeability to O_2_ and CO_2_ was measured at 23 °C and 0% RH, that to H_2_O was measured at 38 °C and 100% RH, and that to N_2_ was measured at 20 °C and 75% RH. 

Four storage temperatures were used for all the samples, from cool to warm temperature conditions: 5, 15, 25, and 35 °C. The relative humidity values of the air in the storage chambers were 72.3%, 70.8%, 52.9%, and 39.8%, respectively.

In summary, the experiment had a factorial design with the four following factors:

Fruit type: apricot and raisin;
Storage temperature: 5, 15, 25, and 35 °C;
Packaging material: glass and three different films;
Packaging atmosphere: air and nitrogen.
Apricots and raisins were used to confirm the results and conclusions in more than one type of fruit.

### 2.3. Analytical Methods

*Film characterization.* Film composition was analyzed by Fourier-transform infrared spectroscopy with Nicolet Magna 550 FT-IR equipment (Nicolet, Paris, France).

*Film thickness.* Thickness was determined using a Metrotec micrometer (Metrotec S.A., San Sebastian, Spain), with a measure interval between 0 and 199.9 mm and 0.1-mm resolution.

*Film permeability.* Permeability was measured by means of a permeation cell with Mocon equipment (Modern Controls, Inc., Minneapolis, MN, USA): the Permatran W-200 model in the case of H_2_O, the Permatran C-200 for CO_2_, and the Oxtran MS 2/20 model for O_2_. Data on permeability to N_2_ were given by the supplier of the films [27].

*Internal atmosphere analysis.* An analysis of the oxygen levels inside the polypropylene packages was carried out with a Toray LC 750-F gas analyzer (Toray Industries, Inc., Osaka, Japan) [28]. This analyzer determines oxygen concentration using the ion conductivity of a zirconia ceramic cell, with a reference gas on one side and the sample gas on the other. The movement of oxygen ions generates an electromotive force which can be measured to determine the oxygen content, according to the Nernst equation.

Carbon dioxide was determined using a Hewlett Packard 5710A Gas Chromatograph (Hewlett Packard, Palo Alto, CA, USA), with a thermal conductivity detector [29] and a Hewlett Packard 3390A integrator (Hewlett Packard, Palo Alto, CA, USA). Headspace gas within the package was sampled via a connecting septum. The injection volume was 1 μL. The column was a 23% SP-1700 Chromosorb P AW (9 m long and 3.2 mm outside diameter; Supelco, Inc., Bellefonte, PA, USA). Helium was used as the carrier gas at a flow of 1.0 mL/min. The detector, injector, and oven temperatures were 150, 100, and 60 °C, respectively.

Internal atmosphere inside the glass containers was not analyzed, since it was not possible to take samples without affecting headspace composition. 

*Dried fruit analysis.* The SO_2_ content was determined using a method that combined a selective distillation in hydrochloric acid with a selective redox titration of sulfite ions by iodine [30]. The moisture content was measured by drying samples to a constant weight at 70 °C in a vacuum oven. In the case of apricots, texture was assessed by a test of compression–extrusion to measure the maximum force (N/cm^2^) required, with a Lloyd L1000 Universal testing machine (Lloyd Instruments Ltd, Fareham, UK) equipped with a 5000-N Kramer load cell. In the case of raisins, this analysis was not carried out because the variety studied had seeds, which would have distorted the results.

### 2.4. Statistical Analysis

The statistical analysis of data was performed using GraphPad InStat software, version 3.0 (GraphPad Software Inc., San Diego, CA, USA). A comparison of the results obtained for the different samples was done by two-way ANOVA (main effect and storage time) using a Student–Newman–Keuls test (*p* < 0.05). All analyses were performed in triplicate. The effects of temperature, packaging atmosphere. and material were analyzed separately to improve the understanding of how far each contributed to the shelf life.

## 3. Results and Discussion

### 3.1. Effect of Temperature

**Figure 1:**
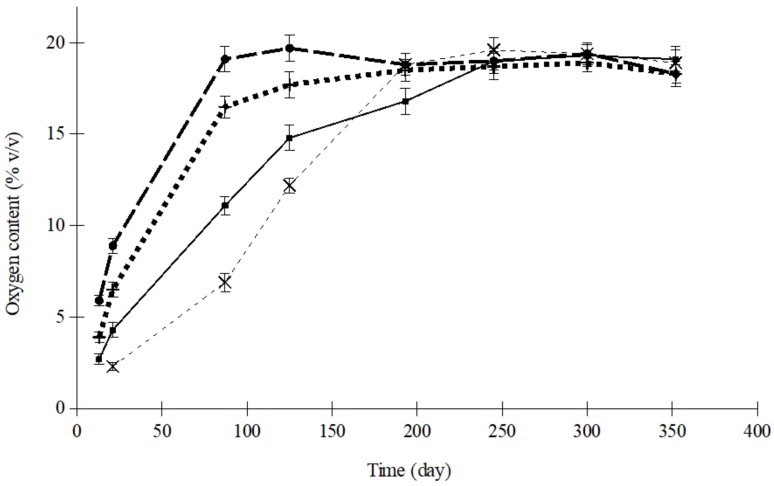
Evolution of O_2_ concentration in trays containing dried apricots sealed with oriented polyamide/polyethylene (OPA/PE) 15/100 film and flushed with N2 (×, 5 °C; ■, 15 °C; +, 25 °C; ●, 35 °C).

**Figure 2:**
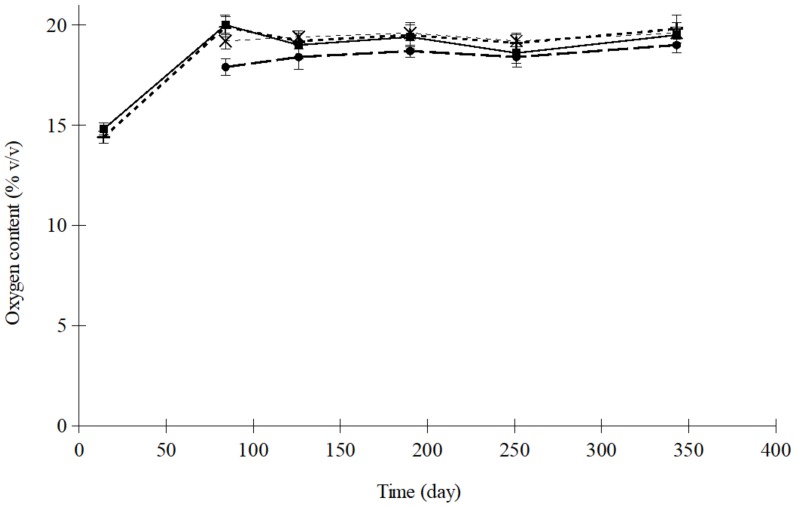
Evolution of O_2_ concentration in trays containing raisins sealed with polyamide/polypropylene (PA/PP) 20/75 film and flushed with N_2_ (×, 5 °C; ■, 15 °C; +, 25 °C; ●, 35 °C).

Both in raisins and apricots, packages sealed with air had similar initial O_2_ and CO_2_ content: 19.6% and 0.036% (*v*/*v*), respectively. When trays were flushed with nitrogen, the other gases were completely removed. Figure 1 illustrates the O_2_ concentration inside the polypropylene trays of dried apricots, sealed with OPA/PE 15/100 film and an initial atmosphere of nitrogen, stored at different temperatures. The case of raisins packaged with PA/PP 20/75 in nitrogen is shown in Figure 2.

In these Figures, it can be observed that, in packages flushed with nitrogen before sealing, oxygen levels increased, regardless of the temperature, until equilibrium with the ambient concentration was eventually reached. The time necessary to arrive at this equilibrium was greatly affected by temperature and, subsequently, it could also be the end of the product shelf life. As the temperature decreased, the length of time the dried apricots and raisin could be stored increased, because O_2_ concentration increased more slowly. 

The oxygen increase inside the tray was likely linked to the permeability of the plastics, which increased as the temperature rose. Thus, the differences observed between both films (Figure 1 and Figure 2), could be due to a different effect of the temperature on film permeability [31]. When taking only the ultimate equilibrium into consideration, temperature presented no significant influence on the final oxygen level reached (*p* > 0.7 in every case studied). Nevertheless, when focusing only on the results obtained before equilibrium was reached, the concentration differences were more significant, particularly with OPA/PE 15/100 (*p* < 0.01).

### 3.2. Effect of Type of Film

**Figure 3:**
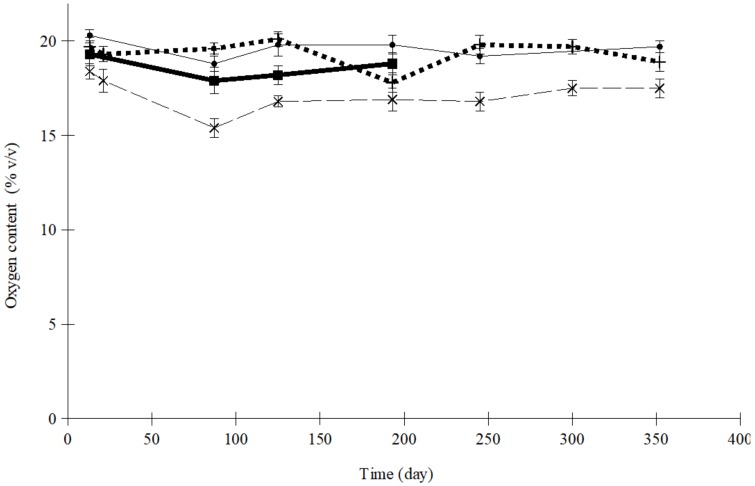
O_2_ concentration in trays containing dried apricots sealed with air (OPA/PE: +, 5 °C; ×, 35 °C; PA/PP: ●, 5 °C; ■, 35 °C).

**Figure 4:**
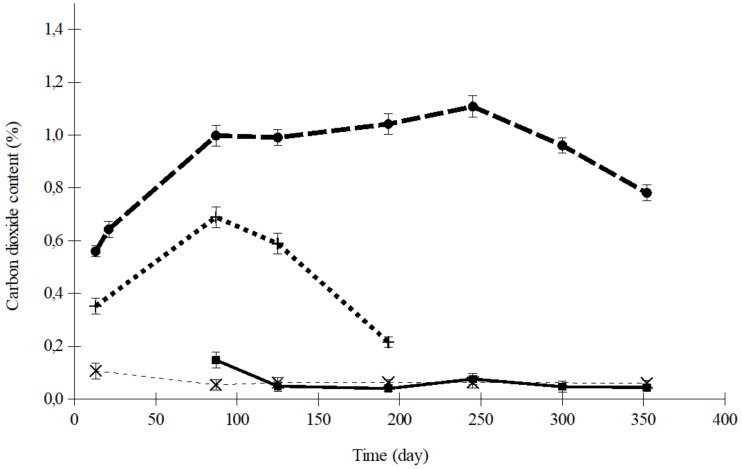
CO_2_ concentration in trays containing dried apricots sealed with air (PA/PP: ×, 5 °C; +, 35 °C; OPA/PE: ■, 5 °C; ●, 35 °C).

Figure 3 shows the variation in O_2_ concentration inside the trays of dried apricots sealed with OPA/PE 15/100 and PA/PP 20/75, at 5 and 35 °C, when the initial atmosphere was air. The corresponding CO_2_ curves for the same samples are shown in Figure 4. The maximum difference between the O_2_ concentrations in these two films, 2.5%, was observed after 87 days (Figure 3). In the case of CO_2_, the maximum difference was 0.826%, after 193 days (Figure 4). In relative terms, the differences between the O_2_ concentrations in the samples were smaller than in the case of CO_2_, probably because the values were much higher for O_2_. Comparing the results at 5 and 35 °C for each polymer, the differences were statistically more significant for CO_2_ (*p* < 0.0001) than for O_2_ (*p* < 0.01).

The experimental data indicate that, when the initial atmosphere was air, as the temperature increased, the O_2_ concentration decreased and the CO_2_ concentration increased inside the trays, regardless of the film composition. Thus, for example, after 245 days, the O_2_ content (% *v*/*v*) in the OPA/PE sealed containers of dried apricots, with air, was 16.8 at 35 °C and 19.8 at 5 °C. The CO_2_ values (% *v*/*v*) were 1.108 at 35 °C and 0.076 at 5 °C. It is likely that this was due to processes that both produce CO_2_ and other gases, and also consume O_2_ during storage, which increased as the temperature rose. These results are in agreement with those found by Ayhan et al. [32] in a study of MAP technology applied to carrots. They stated that the headspace CO_2_ increase was parallel with the oxygen decrease.

In dried fruits, the respiration rates are very low (<1 mg CO_2_/kg·h at 5 °C) compared to their fresh seasonal counterparts, due to the reduced water content and the living status of the tissues [21]. The perishability of dried fruit is very low, with a potential shelf life of more than 16 weeks in air at near optimal temperatures [33]. Despite this, it is crucial to maintain the initial quality parameters of these dried products during the storage period of one year, since these fruits are harvested once a year and the picking period may extend over three weeks and eight weeks for apricots and grapes, respectively. 

Comparing the results found with both polymers at each temperature, no significant differences were found. Consequently, it could be concluded that, rather than plastic film material, temperature was the most potent variable (Figure 3 and Figure 4). An important criterion when selecting a laminated film for food packaging is the permeability ratio of CO_2_:O_2_. A reason why there were no significant differences between the results obtained with the films used in this study could be that all of these materials had quite a similar ratio, between 3.8 and 5.2. Since both the rate of respiration and the permeability of the film are temperature-sensitive, the storage temperature could be the most determinant factor within this narrow range of permeability ratio.

The variation in CO_2_ concentration presented a maximum value which was especially noticeable at 35 °C (Figure 4). According to Fishman et al. [34], this is an intrinsic characteristic of the CO_2_ concentration curve in films more permeable to CO_2_ than to O_2_ (Table 1). This maximum was achieved as a result of the two processes determining its concentration: food oxidation and the gaseous exchange through the plastic barrier. These two processes can affect the organoleptic quality of the product, since the loss of water and other gases would increase the firmness of the product so much that it would be no good for consumption. In addition, oxidation affects the color of the dried fruit. These maximums in CO_2_ content were also found by Kim et al. [35] in salad savoy packages with different films of selected O_2_ transmission rates. Raisins and dried apricots are especially suitable for mold contamination and subsequent toxin production during storage periods. Some species of mold can produce mycotoxins at high temperatures. Moisture, temperature, and oxygen concentration have a crucial effect on fungal proliferation and mycotoxin biosynthesis. Sulfite prevents the growth of undesirable molds in dried fruits. The volatilization of SO_2_ also affects the level retained for antimicrobial action.

### 3.3. Effect of Initial Atmosphere

**Figure 5:**
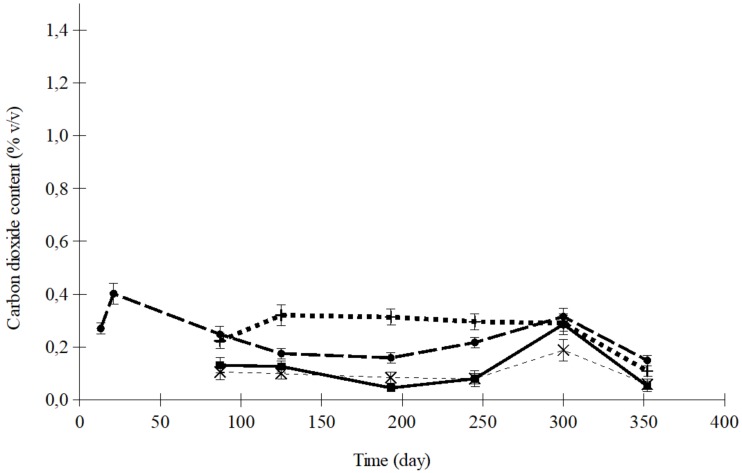
CO_2_ concentration in trays containing dried apricots sealed with OPA/PE 15/100 film and flushed with N_2_ (×, 5 °C; ■, 15 °C; +, 25 °C; ●, 35 °C).

The CO_2_ concentration in trays containing dried apricots stored at different temperatures, flushed with nitrogen, and then sealed with OPA/PE 15/100 film, is shown in Figure 5.

The effect of temperature on the CO_2_ content was much more significant in air than in nitrogen (*p* < 0.0001 air, and *p* < 0.01 nitrogen). 

In relation to nitrogen flushing before sealing, the results showed a very significant influence on the variation in O_2_ content, at every temperature, both in apricots and raisins, with a 99.9% confidence level. The effect of the initial atmosphere was not statistically significant on the changes in the concentration of CO_2_, during 12 months of storage.

### 3.4. Quality of the Dried Fruit

In determining the quality of the produce, the key indicators are texture, moisture, and SO_2_ level. 

Temperature had an important effect on the moisture loss in the case of plastic trays; as the temperature increased, the moisture decreased. Thus, for example, after 354 days, the moisture content in dried apricots stored in OPA/PE containers, with air, was 10.9 ± 0.3 g water/100 g dry solid at 35 °C and 27.9 ± 0.2 at 5 °C. This was not observed in glass containers, where even a slight increase in water content was noticed at the end of the storage period at 35 °C, with 32.1 ± 0.3 and 25.3 ± 0.4 g water/100 g dry solid found in dried apricots and raisins, respectively. Initial atmosphere had no significant effect on moisture evolution. After 344 days, the moisture of raisins stored in trays sealed with PA/PP 20/75, at 5 °C, was 25.2 ± 0.4 and 25.6 ± 0.3 g water/100 g dry solid, when the initial atmosphere was air and nitrogen, respectively. The results corresponding to the samples stored at 35 °C were 12.2 ± 0.4 and 13.2 ± 0.3 g water/100 g dry solid, with air and nitrogen, respectively.

The firmness of dried apricot only underwent a significant increase at the higher temperatures in polypropylene trays sealed with films, since textural changes during storage are generally caused by changes in moisture. At the final storage period, only the texture of samples stored in films at 35 °C was significantly different from the others and reached the value of 100 ± 10 N/cm^2^, both with air and nitrogen. At 354 days, the value found in samples stored in glass was 43 ± N/cm^2^, not significantly different from the initial value (38 ± N/cm^2^).

An important loss in SO_2_ content was observed during the storage of dried apricots and raisins, especially when temperature increased. At 25 and 35 °C, samples contained less than 20% of the initial SO_2_ at the end of the storage period. Neither the packaging material nor the initial atmosphere had a significant effect on SO_2_ concentration changes.

## 4. Conclusions

This study confirmed that, when semipermeable polypropylene packages containing dried apricots and raisins are flushed with nitrogen before sealing, the O_2_ level in the headspace increases during the initial months of storage until the outside O_2_ concentration is reached; as the temperature increases, the time to reach this level decreases. The CO_2_ concentration increases over time, regardless of whether the initial atmosphere is air or nitrogen; as the temperature increases, so does the concentration. This paper also showed that the different plastic films and initial gases used in this study have no significant effect on the quality of the stored dried fruit, due to the inevitable return to outside atmospheric conditions within the headspace.

## Figures and Tables

**Table 1:** Properties of the films used to seal the polypropylene trays.

Film	Thickness (m)	Permeability O_2_ (cm^3^/m^2^ Day)	Permeability CO_2_ (cm^3^/m^2^ Day)	Permeability H_2_O (g/m^2^ Day)	Permeability N_2_ (cm^3^/m^2^ Day)
OPA/PE 15/100	115	83	428	3.6	9
PA/PP 20/75	95	58	237	5.5	40
PA/PE 20/70	90	59	225	5.3	18

OPA—oriented polyamide; PE—polyethylene; PA—polyamide; PP—polypropylene.

## References

[1] Weinberg Z.G., Yan Y., Chen Y., Finkelman S., Ashbell G., Navarro S. (2008). The effect of moisture level on high-moisture maize (*Zea mays* L.) under hermetic storage conditions-in vitro studies. J. Stored Prod. Res..

[2] Conte A., Angiolillo L., Mastromatteo M., Del Nobile A., Muzzalupo I. (2013). Technological options of packaging to control food quality. Food Industry.

[3] Miranda G., Berna À., González R., Mulet A. (2014). The storage of dried apricots: The effect of packaging and temperature on the changes of texture and moisture. J. Food Process. Preserv..

[4] Ranđelović D., Lazić V., Tepić A., Mošić I. (2014). The influence of packaging materials protective properties and applying modified atmosphere on packed dried apricot quality changes. Hemijska Industrija.

[5] Mgaya-Kilima B., Remberg S.F., Chove B.E., Wicklund T. (2015). Physicochemical and antioxidant properties of roselle-mango juice blends; effects of packaging materials, storage temperature and time. Food Sci. Nutr..

[6] Van Bree I., De Meulenaer B., Samapundo S., Vermeulen A., Ragaert P., Maes K.C., De Baets B., Devlieghere F. (2010). Predicting the headspace oxygen level due to oxygen permeation across multilayer polymer packaging materials: A practical software simulation tool. Innov. Food Sci. Emerg. Technol..

[7] Girling P.J., Coles R., McDowell D., Kirwan M.J. (2003). Packaging of food in glass containers. Food Packaging Technology.

[8] Balzarotti S., Maviglia B., Biassoni F., Ciceri M.R. (2015). Glass vs. plastic: Affective judgments of food packages after visual and haptic exploration. Procedia Manuf..

[9] Bhunia K., Sablani S.S., Tang J., Rasco B. (2013). Migration of chemical compounds from packaging polymers during microwave, conventional heat treatment and storage. Compr. Rev. Food Sci. Food Saf..

[10] Nayik G.A., Muzaffar K. (2014). Developments in packaging of fresh fruits—Shelf life perspective: A review. Am. J. Food Sci. Nutr. Res..

[11] Singh R., Giri S.K., Kulkarni S.D. (2013). Respiratory behavior of turning stage mature tomato (*Solanum lycopersicum* L.) under closed system at different temperature. Croat. J. Food Sci. Technol..

[12] Gordon J., Davis E.A., Taub I.A., Singh R.P. (1998). Biochemical processes: Carbohydrate instability. Food Storage Stability.

[13] Bou-Maroun E., Loupiac C., Loison A., Rollin B., Cayot P., Cayot N., Marquez E., Medina A.L. (2013). Impact of preparation process on the protein structure and on the volatile compounds in *Eisenia foetida* protein powders. Food Nutr. Sci..

[14] Oliveira M., Abadias M., Usall J., Torres R., Teixidó N., Viñas I. (2015). Application of modified atmosphere packaging as a safety approach to fresh-cut fruits and vegetables—A review. Trends Food Sci. Technol..

[15] Mangaraj S., Goswami T.K. (2009). Modified Atmospheric Packaging of Fruits and Vegetables for Extending Shelf-Life: A Review. Fresh Prod..

[16] Castellanos D.A., Cerisuelo J.P., Hernandez-Muñoz P., Herrera A.O., Gavara R. (2016). Modelling the of O_2_ and CO_2_ concentrations in MAP of a fresh product: Application to tomato. J. Food Eng..

[17] Mudau A.R., Soundy P., Araya H.T., Mudau F.N. (2018). Influence of Modified Atmosphere Packaging on Postharvest Quality of Baby Spinach (*Spinacia oleracea* L.) Leaves. Hortscience.

[18] Fu M., Xiao G., Wu J., Chen Y., Yu Y., Chen W., Xu Y. (2016). Effects of Modified Atmosphere Packaging on the Quality of Dried Lemon Slices. J. Food Process. Preserv..

[19] Zhang M., Meng X., Bhandari B., Fang Z., Chen H. (2014). Recent Application of Modified Atmosphere Packaging (MAP) in Fresh and Fresh-Cut Foods. Food Rev. Int..

[20] Bodbodak S., Moshfeghifar M., Siddiqui M.W. (2016). Advances in controlled atmosphere storage of fruits and vegetables. Eco-Friendly Technology for Postharvest Produce Quality.

[21] Kader A.A., Kader A.A. (2002). Postharvest Biology and Technology: An Overview. Postharvest Technology of Horticultural Crops.

[22] Kurtzman C.P., James S.A., Blackburn C. (2006). Food Spoilage Microorganisms. Food Science Technology and Nutrition.

[23] Miranda G., Berna À., Salazar D., Mulet A. (2009). Sulphur dioxide during dried apricot storage. LWT Food Sci. Technol..

[24] Miranda G., Berna À., Bon J., Mulet A. (2011). Modeling of the process of moisture loss during the storage of dried apricots. Food Sci. Technol. Int..

[25] Sanjuán N., Bon J., Bermejo M.V., Tarrazó J., Mulet A., Ortega E., Parada E., Fito P. (1996). Influencia de las condiciones de almacenamiento en la calidad de orejones de albaricoques deshidratados. Anales del I Congreso Iberoamericano de Ingeniería de Alimentos.

[26] García J. (1996). Deshidratación de Vegetales por Aire Caliente: Efecto de la Contracción y la Porosidad en el Secado de la Coliflor.

[27] Aucejo S. (Spain. 2000). Estudio y Caracterización del Efecto de la Humedad en las Propiedades Barrera de Estructuras Poliméricas Hidrofílicas. Ph.D. Thesis.

[28] Mullan M., McDowell D., Coles R., McDowell D., Kirwan M.J. (2003). Modified atmosphere packaging. Food Packaging Technology.

[29] Noh J.K.M. (USA. 2005). Effect of Chitosan and Water Soluble Chitosan Coatings on Quality of Small Fruits. Master’s Thesis.

[30] DeVries J.W., Ge H., Ebert F.J., Magnuson J.M. (1986). Analysis for total sulfite in foods by using rapid distillation followed by redox titration. J. Assoc. Off. Anal. Chem..

[31] Mo C., Yuan W., Lei W., Shijiu Y. (2014). Effects of Temperature and Humidity on the Barrier Properties of Biaxially-oriented Polypropylene and Polyvinyl Alcohol Films. J. Appl. Packag. Res..

[32] Ayhan Z., Efiturk O., Tafi E. (2008). Effect of modified atmosphere packaging on the quality and shelf life of minimally processed carrots. Turkish J. Agric. For..

[33] Kader A.A., Preece J.E., Read P.E. (1993). Postharvest Handling. The Biology of Horticulture: An Introductory Textbook.

[34] Fishman S., Rodov V., Peretz J., Ben-Yehoshua S. (1995). Model for gas exchange dynamics in modified-atmosphere packages of fruits and vegetables. J. Food Sci..

[35] Kim J.G., Luo Y., Gross K.C. (2004). Effect of package film on the quality of fresh-cut salad savoy. Postharvest Biol. Technol..

